# A Psycho-Genetic Study of Hedonic Responsiveness in Relation to “Food Addiction”

**DOI:** 10.3390/nu6104338

**Published:** 2014-10-16

**Authors:** Caroline Davis, Natalie J. Loxton

**Affiliations:** 1 School of Kinesiology & Health Sciences, 343 Bethune College, York University, 4700 Keele Street, Toronto, ON M3J 1P3, Canada; 2 School of Applied Psychology, Griffith University, 176 Messines Ridge Road Mt Gravatt, Queensland 4122, Australia; E-Mail: n.loxton@griffith.edu.au

**Keywords:** food addiction, hedonic responsiveness, *mu* opioid receptor, A118G

## Abstract

While food addiction has no formally-recognized definition, it is typically operationalized according to the diagnostic principles established by the *Yale Food Addiction Scale*—an inventory based on the symptom criteria for substance dependence in the DSM-IV. Currently, there is little biologically-based research investigating the risk factors for food addiction. What does exist has focused almost exclusively on dopaminergic reward pathways in the brain. While brain *opioid signaling* has also been strongly implicated in the control of food intake, there is no research examining this neural circuitry in the association with food addiction. The purpose of the study was therefore to test a model predicting that a stronger activation potential of opioid circuitry-as indicated by the functional A118G marker of the *mu*-opioid receptor gene-would serve as an indirect risk factor for food addiction via a heightened hedonic responsiveness to palatable food. Results confirmed these relationships. In addition, our findings that the food-addiction group had significantly higher levels of hedonic responsiveness to food suggests that this bio-behavioral trait may foster a proneness to overeating, to episodes of binge eating, and ultimately to a compulsive and addictive pattern of food intake.

## 1. Introduction

A recognition that compulsive overeating can foster clinically significant emotional and social impairment in some individuals prompted the American Psychiatric Association (APA) to designate Binge Eating Disorder (BED) a *bona fide* mental illness in the “Feeding and Eating Disorders” chapter of the recently published fifth edition of their *Diagnostic and Statistical Manual* (DSM-5) [1]. Concurrently, the DSM-5 also recognized, for the first time, the existence of Non-Substance-Related Disorders in its chapter on “Substance-Related and Addictive Disorders”, although Gambling was the only disorder listed in this category at the time of publication [2].

The shift in psychiatric thinking, reflected in both these chapters of the DSM-5, may have contributed to a burgeoning clinical and preclinical interest in the topic of *food addiction*. This putative condition is unique, however, by straddling both *substance-related* and *non-substance-related* addiction disorders. On the one hand, there is a growing acknowledgement that many processed foods-specifically those whose palatability is enriched by high levels of added sugar, fat, and salt-have properties similar to substances like cocaine, nicotine, and alcohol in their ability to perturb brain reward mechanisms (see [3,4]). Moreover, when taken in excess they can foster neuro-adaptations that promote compulsive intake, dependence, and cravings, in the same manner as addictive drugs. On the other hand, the very act of eating can be viewed as a potentially addictive behavior because of its ability to arouse all the senses in a highly pleasurable way, from the sounds and aromas of cooking, to the aesthetic appeal of visually colorful and attractively arranged food. Even the tactility of certain foods in one’s mouth can be highly rewarding before they are even ingested.

Interestingly, some public-perception evidence suggests that the notion of food addiction is more vulnerable to stigmatization than that of smoking or alcoholism, and that it tends to be viewed as a behavioral rather than a substance-related disorder [5]. In other words, food addiction is often perceived as a “problem of the mind” where the causes focus on eating as a personal choice and a coping mechanism for alleviating personal unhappiness. According to this view, the pathology is the compulsive overeating; it is *not* vitally related to the addictive quality of certain foods. However, other recent experimental research found that when a random selection of adult participants was presented with a food-addiction model of obesity with a focus on causal biological mechanisms, stigmatization and blame towards overweight individuals was reduced compared to ratings from another group of participants who were given a non-addiction model of obesity. In the former group, there was also a reduction in the view that obese individuals are mentally impaired, and a decrease in the participants’ fear of personal weight gain [6].

### 1.1. Hedonic Responsiveness and Capacity for Reward

Hedonic responsiveness is a highly heritable trait reflecting individual differences in the motivation to seek out rewarding stimuli in one’s environment, and in the capacity to experience pleasure from these events [7]. *Natural* rewards comprise all those incentives important for our survival like eating, reproduction, and mastery. Attempts to understand the biological basis of hedonic responsiveness has focused largely on the sensitivity, or arousability, of the mesocorticolimbic dopamine pathways [7]. A chronic attenuation in the ability to experience reward–duly named *anhedonia*-was first described clinically in the late 19th century as a core feature of many psychiatric disorders including depression, schizophrenia, and drug withdrawal [8]. It is generally agreed that *hypo*-functioning of brain reward circuitry can be an innate human characteristic determined by a concatenation of genetic effects that jointly contribute to a low activation potential [9]. However, such a state can also be induced by excessive stimulation of dopaminergic pathways via potent dopamine agonists like substances of abuse and/or by chronic stress-factors which tend to foster a down-regulation and diminished responsiveness of the system [10].

More recently, the bipolar opposite of anhedonia–high *reward sensitivity*-has been associated with risk for binge eating and other impulse control disorders, based on the argument that those with a strong motivation for reward are more likely to engage in pleasurable behaviors with insufficient restraint compared to their more anhedonic counterparts [11,12,13]. Foods consumed during binges are almost always highly caloric and hyper-palatable [14], suggesting an important role for neural circuitry that regulates hedonically-driven eating in the risk profile for compulsive overeating. Hedonic responsiveness to food is a specific manifestation of the more general trait described above, and reflects the degree of desire to eat, and the pleasure derived from foods which are highly palatability, and fresh and attractive in appearance. Consequently, one with heightened capacity for food reward is typically driven to eat even in the absence of hunger or caloric need [15], and experiences strong food cravings [16].

### 1.2. The Biological Basis of Food Addiction

To date, there is a dearth of biologically-based research investigating the risk factors for food addiction. What does exist has focused almost exclusively on dopaminergic reward pathways in the brain. For instance, a recent study demonstrated that adults with food addiction had a significantly higher score on a composite genetic index of dopamine signaling strength compared to their age- and weight-equivalent counterparts [16]. A neuroimaging study also found that reward-circuitry activation in the amygdala and caudate nucleus, in response to a food cue, was positively associated with food-addiction symptoms in a group of adult women [17]. Together these finding mesh with other psycho-behavioral evidence that food addiction [18], like BED, is a high reward-responsive phenotype of obesity [9]. There is also preliminary support for the view that some cases of food addiction may be a more pathological and compulsive sub-type of BED rather than a distinctly different clinical entity [19]. In addition, the co-occurrence of food addiction with bulimia nervosa (BN) has been related to more severe eating pathology [20]. However, there are also individuals with an apparent food addiction who display elevated BMI (Body Mass Index) and clinical impairment despite not meeting criteria for BN or BED, suggesting that cases of food addiction are not always characterized by episodes of binge eating [20]. This recent evidence also meshes with findings from two earlier studies where only half the obese adults who were diagnosed with food addiction met criteria for BED [18,21].

#### Brain Opioid Pathways and Food Reward

While *opioid signaling* in the striatal area of the brain has also been strongly implicated in the control of food intake, currently there is no research examining the influence of this neural circuitry in the risk profile for food addiction. A wealth of prior related research indicates, however, that activation of the *mu*-opioid receptor (MOR) in the nucleus accumbens selectively promotes hedonically-driven eating in the form of increasing consumption of sweet and fatty foods [22,23]. In addition, signaling via accumbens MOR appears to regulate learned food preferences, and increased levels have been found to promote binge-like consumption of palatable and preferred foods [24]. Conversely, *mu*-opioid antagonists tend to reduce hedonic response to, and the consumption of, tasty foods in binge-eating and overweight adults [25]. There is also evidence that over-stimulation of MOR from excessive consumption of highly palatable foods may prompt down-regulated opioid signaling due to long-term changes in receptor function [26]. On the other hand, a recent clinical study found that weaker opioid activity was associated with greater hedonic-related eating, greater intake of high-calorie food, and greater bingeing, although these findings are somewhat compromised because assessment was made using an indirect measure of activity [27]. In summary, converging research indicates that central opioid activity is likely involved in addictive symptoms related to palatable-food intake including bingeing, cravings, and withdrawal despite the direction of causality being uncertain [27].

Of the many genetic variants identified on the MOR gene (OPRM1), the A118G (rs1799971) single nucleotide polymorphism (SNP), located in the coding region of exon 1, has been the most widely studied, especially in relation to drug addiction Although the exact mechanisms remain unclear, an *in vitro* study has demonstrated that the minor G allele causes a threefold increase in binding affinity for endogenous beta-endorphins and it augments G protein-coupled potassium activation [28]. Recent *in vivo* evidence also supports the notion G allele is indeed a “gain-of-function” for those possessing this minor allele [29]. For instance, one study reported a greater prevalence of the G allele in both alcoholic and opioid addicts in India compared to the general population [30], similar to findings from an earlier Swedish study [31]. A group of heavy drinkers carrying the G allele also reported greater hedonic responses to alcohol compared to their counterparts with the AA genotype, although they did not differ on the sedative and aversive effects of alcohol [32]. Not all studies, however, have found such associations in drug-addiction research [33,34].

Genetic association studies have also examined *dimensional* symptoms associated with the clinical presentation of addictive behaviors. For example, adolescent carriers of the G allele had more alcohol-related problems and reward-focused drinking motives than those without this allele [35]. Similarly, as indicated by the activation of mesocorticolimbic brain structures, adult G carriers exhibited greater dose-dependent responsiveness to the reinforcing effects of alcohol, and a greater sensitivity to alcohol cues [36,37].

There is further evidence that variation in OPRM1 function predicts sensitivity to *natural* rewards. Among infant monkeys, G allele carriers formed stronger attachment bonds with their mothers and showed greater distress during periods of maternal separation [29]. Relatedly, human G carriers have demonstrated a greater social hedonic capacity as indicated by an increased tendency to engage in affectionate relationships and a greater display of pleasure in social situations [38]. In addition, we found, for the first time, *mu*-receptor genotype differences in relation to the liking of sweet and fatty foods with the homozygous GG group reporting higher food-preference ratings compared to the other two groups [18]. Different, however, from other studies where the GG and GA genotype groups are typically combined in the statistical analyses, our findings suggested a recessive form of transmission in which two copies of the G allele are required to convey the effect.

### 1.3. The Current Study

While food addiction has no formally-recognized definition, it is typically operationalized according to the diagnostic principles established during the development of the *Yale Food Addiction Scale* (YFAS) [39]—a self-report inventory based on the symptom criteria for substance dependence in the DSM-IV [40]. In general, it is characterized by chronic, escalating, and compulsive overeating, often in the form of binge episodes, as confirmed by its considerable co-morbid overlap with BED [18,21].

**Figure 1:**
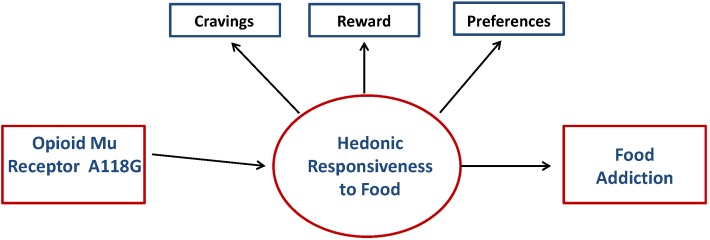
Model predicting that the OPRM1 A118G genetic marker will relate to the hedonic-responsiveness composite variable, which in turn will be positively associated with YFAS symptom scores.

The current study is the first to examine a biological indicator of brain opioid functioning in the risk profile for YFAS food addiction. Specifically, the purpose was to test the indirect-effects model illustrated in Figure 1. Specifically, we predicted that a stronger activation potential of opioid circuitry in the common reward pathway-as indicated by the GG polymorphism of the functional A118G marker of MOR-would serve as a risk factor for food addiction. The mechanism of conduction was hypothesized to be an indirect relationship via a heightened hedonic responsiveness to palatable food. Specifically, the GG genotype would be associated with greater hedonic responsiveness, modeled as a composite variable with three separate indicators–viz. hedonic eating, food cravings, and a preference for sweet and fatty foods. In turn, hedonic responsiveness was predicted to correlate positively with symptoms of food addiction as indicated by scores on the YFAS.

## 2. Methods

### 2.1. Participants

One hundred and forty-five adults (women: 100; men: 45) between the ages of 25 and 47 years took part in the study. The ethnic distribution of the sample was 80% Caucasian, 16% African descent, and 4% other. Participants were recruited from posters placed at public institutions requesting volunteers for a study of eating behaviors. Advertisements were also placed in local newspapers and online sites. Participants were required to be fluent in English, and to have lived in North America for at least five years prior to their enrolment to ensure a relatively uniform food environment for a substantial period of time preceding enrolment in the study.Women were also required to be pre-menopausal as determined by the self-reporting of regular menstrual cycles, and not to have had a pregnancy within the previous six months. Exclusion criteria included a current (or history of) diagnosis of any psychotic disorder or substance abuse. Those with a serious medical/physical illness such as cancer or heart disease were also excluded, as well as those taking medication that affected appetite (e.g., stimulant drugs). The procedures employed in this study were approved by the institutional Research Ethics and were carried out in accordance with the Declaration of Helsinki.

### 2.2. Measures

#### 2.2.1. Genotyping

DNA extraction from whole blood was completed by the non-enzymatic, high-salt procedure as described by Lahiri and Nurnberger [41]. We tested the functional A118G single nucleotide polymorphism (SNP), which causes a missense amino acid change from an aspartate residue to an asparagines residue, thus potentially removing an *N*-glycosylation site [42]. This SNP was genotyped using commercially available genotyping assays (Applied Biosystems Inc., Foster City, CA, USA). Genomic DNA (20 ng) was amplified in 10-μL reactions by polymerase chain reaction with the following conditions: 95 °C 10 min, followed by 50 cycles of 92 °C 15 s, 60 °C 1min. The Allelic Discrimination Program on ABI7000 Prism Sequence Detection System was used to determine the genotypes of each individual. Genotypes were tested for fitness to Hardy-Weinberg Equilibrium using Haploview version 4.2 (Broad Institute, Cambridge, MA, USA) [43].

#### 2.2.2. Self-Report Questionnaires

**Food Addiction** was diagnosed using the YFAS. This measure has high convergent validity with other measures of eating pathology, especially binge eating, and may therefore be a useful tool to identify individuals with addictive tendencies towards food [39]. This 25-item scale was designed to operationalize food addiction according to the 7 symptoms of substance dependence listed in the DSM-IV, and modified for eating behaviors. The YFAS provides both a qualitative (binary) and quantitative method of scoring. Similar to the DSM substance-dependence criteria, a diagnosis of food addiction is given if the respondent experienced three or more symptoms over the past year, and if the “clinically significant impairment” criterion is met. The dimensional score is obtained by summing the number of symptoms endorsed, and therefore it can range from 0 to 7. For this sample, Cronbach’s alpha coefficient for the symptom score was 0.78.

**Preference for High Fat and High Sugar Foods** was assessed by the *Food Preference Questionnaire* [44], which is a 72-item scale designed as a 2 (FAT: high *vs.* low) × 3 (CARBOHYDRATE: high simple, high complex, low carbohydrate/high protein) measure of preference for various kinds of macronutrients. Respondents indicate their preference for each food on a nine-point Likert scale. The *High Fat and High Sugar Preference* score is the mean of 12 fatty and sugary food-item ratings (e.g., chocolate layer cake, and pecan pie). The authors report good reliability and validity of these measures, and the alpha coefficient for this scale in our study was 0.81.

**Hedonic Eating** was assessed by the *Power of Food Scale* [45], which is a 21-item questionnaire that reflects individual differences in the appetitive responsiveness to food in environments with an abundance highly palatable food, independent of a person’s actual consumption of these foods. In other words, it differentiates the motivation and appetitive drive to obtain food from the tendency to (over)eat food. Cronbach’s alpha coefficient in this study was 0.96.

**Food Cravings** were assessed by the *Food Craving Questionnaire–Trait* [46]. This 39-item scale reflects both the physiologically and psychologically aspects of food cravings—for example, as feelings of hunger, preoccupation with food, and lack of control. The alpha coefficient was 0.97.

### 2.3. Procedures

In order to confirm initial eligibility, a telephone pre-screening was conducting with those who indicated interest in participating in the study. On the appointment day, a structured, face-to-face, clinical interview was also carried out to re-confirm eligibility, after which informed consent and all relevant demographic information were obtained. Height and weight were measured with the participant standing in stocking feet and wearing light indoor clothing. A venous blood sample was taken at the hospital laboratory, and the questionnaire package was completed at home and returned at a later date.

### 2.4. Statistical Analyses

Hardy-Weinberg equilibrium and linkage disequilibrium were assessed using a chi-squared test through Haploview, version 4.2 (Broad Institute, Cambridge, MA, USA) [43]. Differences between OPRM1 A118G genotypes and continuous level variables were assessed in IBM SPSS Statistics for Mac, Version 22 (IBM Corp., Armonk, NY, USA) using Analysis of Variance (ANOVA) procedures. In order to test whether there was an indirect effect of the A118G marker and the food-addiction symptom score via hedonic responsiveness, the procedures described by Hayes and Preacher [47] were followed. This approach allows the use of multi-categorical independent variables, and tests the significance of the indirect effect using bias-corrected bootstrapping. The SPSS “MEDIATE” macro-developed to accompany the paper by Hayes and Preacher [47]—was employed to test the significance of the direct effects. As there are three genotype groups, indicator coding was tested with the heterozygous GA set as the reference group (A similar pattern of results was found when setting the GG allele group as the reference group). This approach to testing indirect effects calculates a cross-product of path *a* (the association between the predictor variable, *i.e.*, genotype group and the intermediary variable *i.e.*, hedonic responsiveness) and path *b* (the association between the intermediary variable and the outcome variable, *i.e.*, symptoms of food addiction). In this study, the bias-corrected bootstrap confidence intervals (*n =* 1000) were set at 95%, and were used to assess the significance of indirect effects. Because there are three genotype groups, there are two *a* paths (GG *vs.* GA and AA *vs.* GA) and subsequently, two tests of indirect effects. An absence of zero in the confidence interval indicates significant indirect effects.

## 3. Results

### 3.1. Descriptive Statistics

Table 1 presents the allele and the genotype frequencies for the functional A118G SNP, listed separately for the food-addiction and the non-food addiction groups. Results also confirmed that this marker was in Hardy-Weinburg equilibrium. Previous research indicates that the allele frequencies for this marker tend to be somewhat different across ethnic groups [30]. Since a large proportion of the current sample is Caucasian, however, and because the sample is not sufficiently large to stratify by ethnicity, we have assessed all observations together. It can be seen that the frequency of the G allele in our full sample is very similar to other Caucasian samples summarized in Deb and colleagues’ review [30], and in a previous study using a similar sample [12].

**Table 1:** Allele and genotype frequencies (with genotype percent within each diagnostic group) for the OPRM1 A118G SNP, listed separately for the food-addiction (*n =* 25) and non-food-addiction (*n =* 114) groups.

Group	Allele		Genotype		
	G	A	GG	GA	AA
**Food Addiction**	9	41	2 (8%)	5 (20%)	18 (72%)
**Non-Food Addiction**	44	184	5 (4.4%)	34 (29.8%)	75 (65.8)

Note: One observation in the food-addiction group and five observations in the non-food-addiction group (4.1% in total) had missing data for the A118G SNP.

The three hedonic-responsiveness variables (*i.e.*, food cravings, hedonic eating, and high fat/sugar preference) were moderately to highly inter-correlated as expected. A composite score was therefore calculated using Principal Component Analysis. The extracted component accounted for 66% of variance in the three scales, and all three loaded strongly on this factor (loadings ranging between 0.52 and 0.93). This approach resolves problems associated with multi-collinearity that would adversely affect subsequent analyses if the three variables were added to the model individually. It also increases scale reliability [48].

Table 2 shows means and standard deviations for age, BMI, hedonic-responsiveness (factor score) and food-addiction symptoms. One-way ANOVA procedures found no significant differences between the genotype groups on age, BMI, or food-addiction symptom scores. There was, however, a significant difference in hedonic-responsiveness. *Post hoc* comparisons, using the Least Significant Difference procedure, found that both the GG and the AA groups had significantly higher hedonic-responsiveness scores than the GA group (GG *vs.* GA, *p =* 0.026; AA *vs.* GA, *p* = 0.004), but they did not differ from each other (GG *vs.* AA, *p =* 0.368). Hedonic responsiveness was also positively associated with the YFAS symptom score (*r =* 0.68, *p <* 0.001). A binomial logistic regression was also performed to assess the association between hedonic responsiveness and YFAS diagnosis. As predicted, higher composite scores were associated with a greater likelihood of meeting diagnosis for food addiction (*B* = 1.89, *Bse* = 0.36, Wald = 28.22, *p <* 0.001). However, given the low frequency of participants in the food addiction x genotype groups, it was more appropriate statistically to use the YFAS symptom score as the criterion in the subsequent analyses.

**Table 2:** Means, standard deviations, and minima and maxima for all quantitative variables, listed separately for the three genotypes.

Variable	GG	GA	AA	F
**Age**	31.9 (6.5:26–44)	33.2 (6.2: 25–45)	32.6 (6.6:25–47)	0.22
**BMI**	31.1 (8.0:19.5–40.9)	32.2 (8.6: 19.6–51.4)	33.9 (8.4:19.0–60.1)	0.82
**Hedonic Responsiveness**	0.5 (0.9:−0.6–1.6) ^a^	−0.4 (0.8:−2.4–1.4)	0.1 (1.0:−2.4–2.5) ^a^	5.31 **
**YFAS Symptom Score**	3.1 (2.1:1–7)	2.2 (1.7:0–6)	2.9 (2.0:0–7)	1.95

Note: ** *p* <0.01, ^a^ GG and AA groups were both significantly higher than the GA group.

A test of sex effects, using independent t-test procedures, indicated no significant group differences on the hedonic responsiveness composite score or the YFAS Symptom score.

### 3.2. Indirect Effects

**Figure 2:**
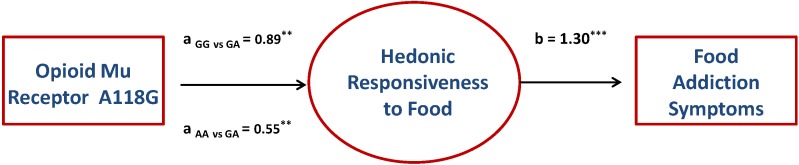
Indirect effects model of the relationship between A118G genotypes, hedonic responsiveness to food, and YFAS symptom scores. Unstandardized coefficients are presented and tested for significance with 95% confidence Intervals calculated using the bias-corrected bootstrap method (1000 samples). a = unstandardized genotype to hedonic coefficients, b = unstandardized hedonic eating to YFAS coefficient Subscripts refer to relative indirect paths. GA is the reference group. ** *p <* 0.01; *** *p <* 0.001.

Given the significant association between genotype groups and the hedonic-responsiveness factor score, and because the latter was also significantly associated with YFAS symptom scores, tests of indirect effects were performed to assess whether hedonic responsiveness acted as a potential mediating pathway between the A118G marker and food addiction. The direct effect of genotype group and food addiction (in the absence of the “mediating” variable) was not significant. It should be noted, however, that tests of indirect effects can be performed in the absence of a direct association between a predictor variable and an outcome variable [49,50]. This is particularly so for predictor variables which are quite distal to the outcome variable, as is the case between genetic factors and symptoms of food addiction. Results of the model tested are shown in Figure 2. As the genotype groups are categorical, indicator coding (also known as *dummy* coding) was used in accord with recommendations by Hayes and Preacher [47]. The GG and AA genotypes were tested against the GA genotype. As shown in Table 3, participants with either a GG or AA genotype were higher in hedonic-responsiveness relative to the GA genotype (path a), which in turn was associated with higher YFAS symptom scores (path b). The indirect effects from both GG and AA genotypes (relative to GA) were significantly different from zero. Similar support was found when testing indirect effects on the YFAS diagnosis score as the criterion using the Hayes [50] PROCESS macro (Indirect Effect _GG *vs.* GA_ = 1.83, 95% CI = 0.23–3.75; Indirect Effect _AA *vs.* GA_ = 1.13, 95% CI = 0.42–2.00). This model supports the hypothesis that the GG genotype (although rare) is associated with higher food-addiction symptoms via a heightened responsiveness to hedonically-rewarding foods. Unexpectedly, the AA genotype was also associated with greater risk for food addiction via a similar bio-behavioural predisposition Explicitly testing the indirect effect of the AA *vs.* GG allele groups showed there was no difference between these two groups (Indirect Effect = −0.44, 95% CI = −1.56–0.53). Controlling for sex and BMI did not substantively change these results.

**Table 3:** Indirect effects of A118G genotypes on YFAS symptoms scores through hedonic responsiveness.

Polymorphism	Bootstrap Estimate	SE	BC 95% CI Lower	BC 95% CI Upper
AA *vs.* GA	0.71 *	0.23	0.30	1.19
GG *vs.* GA	1.16 *	0.50	0.26	2.21

Note: Based on 1000 bootstrap samples. BC = biased corrected; CI = Confidence Interval, * Indirect effect is significantly different from zero. All effects are reported as unstandardized coefficients.

## 4. Discussion

The results of this study partially supported the model shown in Figure 1, and our prediction that the G “gain-of-function” allele of the A118G marker is associated with high hedonic responsiveness to palatable food. Unlike our previous research, however, where an apparent recessive mode of transmission was found for the G allele and food preferences [12], the current data indicated that although the GG genotype had the highest mean hedonic-responsiveness score, it did not differ significantly from the homozygous AA group. Moreover, the heterozygous GA genotype demonstrated significantly **lower** hedonic responsiveness than either of the two homozygous groups, implying an *over-dominant* (*Over-dominance* refers to a condition where the heterozygous group lies outside the phenotypic range of both homozygous groups, and can be assumed to have lower risk for a potentially deleterious trait—in other words, a higher fitness-than homozygous individuals) effect for this marker. Interestingly, there is considerable evidence of heterozygosity-fitness correlations in the general population, and some believe this occurs because inbreeding increases the level of homozygosity on a genome-wide basis, and is also associated with a decline in fitness-associated traits [51]. Unfortunately, our genetic findings are difficult to verify with other related research since many studies examining the A118G SNP in addiction-related research have assumed a dominant mode of transmission for G, thereby creating a binary A118G variable (viz. GG and GA *vs.* AA) for the purposes of analysis (e.g., [32,52,53]). The appropriateness of such a strategy can now be questioned, not only as a result of the findings from this study, but also on the basis of recent meta-analytic evidence showing an overall significant association of A118G with responsiveness to opioids under a *co-dominant* or *additive* model [54]. As a consequence, future researchers in this area are encouraged to analyze the A118G SNP using three instead of two genotype groups. In addition, given the relatively low frequency of observations in the homozygous (minor allele) G group, it is probable that our study was underpowered to detect significant differences between the GG and AA groups despite the higher mean score in the former. Therefore, research with larger samples is necessary to further test our proposed model and its predicted associations.

Our study results also confirmed that hedonic responsiveness was significantly and positively associated with symptom scores on the YFAS and with YFAS-diagnosed food addiction. These findings support a wealth of accumulating evidence that hedonic brain systems are strongly influential in driving the overconsumption of energy-dense foods [55]. Indeed, an elevated hedonic responsiveness to food may increase risk for overeating by fostering the disproportionate selection of rich and highly-palatable food in one’s daily diet, as well as by hindering attempts at abstaining from such patterns of food intake. For instance, recent preclinical evidence has demonstrated that rats exposed to prolonged and excessive intake of calorically-densefood showed increased reward thresholds to electrical brain stimulation (indicative of a diminished sensitivity to reward) [56], and long-term palatable food intake also led to a decrease in *mu*-opioid mRNA expression in the nucleus accumbens-again indicating a system down-regulation [57].

It has been suggested by some that a diminished reward response tends to foster an increased motivation to compensate for this deficiency by overeating [58,59]. In our view, however, such an explanation is too simplistic, especially in light of the compelling evidence that anhedonia is associated with a depressive demeanor, a diminution in appetite, and a reduced motivation to engage in normally rewarding experiences such as social interaction and parental caregiving [60,61]. A more complete explanation for the relationship between reward sensitivity and food intake is provided by a dual-process model [62]. From an individual-vulnerability perspective, high hedonic responsiveness to food predisposes to elevated food intake, and eating for pleasure beyond caloric need, especially in a food environment with an ubiquitous availability of tasty food. In turn, chronic overstimulation of brain reward circuitry by excessive consumption can down-regulate the activation potential of mesocorticolimbic pathways (as described above) while concurrently enhancing the salience of rich and tasty foods, which creates strong cravings and food-seeking behaviors [62]. The consequent reward-system down-regulation can thereby contribute to the maintenance of overeating and to the relapse proneness following periods of dietary restraint [63]. Indeed, those who are symptomatic for food addiction typically report a poor prognosis in their efforts to normalize their eating behaviors [14].

A particular strength of the current study was an explicit test of the indirect effect of the functional OPRM1 SNP and food addiction via hedonic responsiveness. Specifically, this test supported our proposal of an indirect effect of genetic vulnerability via the “hedonic pull” of highly palatable foods towards more pronounced symptoms of food addiction. This finding is similar to previous indirect effect models examining psychological and behavioral processes as potential pathways from specific genetic profiles to food-addiction diagnosis and risk for obesity [16,64]. As with all putatively causal models, however, prospective data are required to verify these findings.

Despite the significant and novel findings from this research, it is important to draw attention to its limitations. Notably, the genetic findings must be viewed cautiously and strictly as preliminary due to the small number of observations in the GG genotype group relative to the other two groups, and because of the relatively low frequency of individuals in the YFAS food-addiction group. Replication with larger samples will allow for greater confidence in, and reliability of, the findings reported here.

## 5. Conclusions

In summary, the results of this study have demonstrated; in a preliminary way, the relationship between brain opioid signaling strength and human variation in hedonic responsiveness to tasty and highly caloric foods. They have also indirectly implicated opioid activation-potential in the risk for compulsive overeating. There is still; however; insufficient evidence to determine with confidence the mode of transmission of the OPRM1 A118G marker on enhanced responsiveness to opioid agonists like palatable food and various addictive drugs. In addition; further support for the validity of the food-addiction construct is provided by our findings given that the food-addiction group had significantly higher levels of hedonic responsiveness to food—a biobehavioral trait that may foster a proneness to overeating; to episodes of binge eating; and ultimately to a compulsive and addictive pattern of food intake.
